# Upregulation of the solute carrier family 7 genes is indicative of poor prognosis in papillary thyroid carcinoma

**DOI:** 10.1186/s12957-018-1535-y

**Published:** 2018-12-17

**Authors:** Lei Shen, Chunhua Qian, Huimin Cao, Zhongrui Wang, Tingxian Luo, Chunli Liang

**Affiliations:** 1 Department of Thyroid Breast Oncology, Shanghai East Hospital, School of Medicine, Tongji University School of Medicine, No.150 Jimo Road, Shanghai, 200120 China; 2 Department of Endocrinolgy and Metabolism, Shanghai Tenth People’s Hospital, Tongji University School of Medicine, Shanghai, China; 3 Department of Thyroid and Breast, Shanghai Tenth People’s Hospital, Tongji University School of Medicine, Shanghai, China

**Keywords:** PTC, SLC7A3, SLC7A5, SLC7A11, Overall survival

## Abstract

**Background:**

The solute carrier (SLC) 7 family genes comprise 14 members and function as cationic amino acid/glycoprotein transporters in many cells, they are essential for the maintenance of amino acid nutrition and survival of tumor cells. This study was conducted to analyze the associations of SLC7 family gene expression with mortality in papillary thyroid carcinoma (PTC).

**Methods:**

Clinical features, somatic mutations, and SLC7 family gene expression data were downloaded from The Cancer Genome Atlas database. Linear regression model analysis was performed to analyze the correlations between SLC7 family gene expression and clinicopathologic features. Kaplan-Meier survival and logistic regression analyses were performed to characterize the associations between gene expression and patients’ overall survival.

**Results:**

Patient mortality was negatively associated with age and tumor size but positively increased cancer stage and absence of thyroiditis in PTC patients. Kaplan-Meier survival analysis indicated that patients with high SLC7A3, SLC7A5, and SLC7A11 expression levels exhibited poorer survival than those with low SLC7A3, SLC7A5, and SLC7A11 expression levels (*P* < 0.05 for all cases). Logistic regression analysis showed that SLC7A3, SLC7A5, and SLC7A11 were associated with increased mortality (odds ratio [OR] 8.61, 95% confidence interval [CI] 2.3–55.91; OR 3.87, 95% CI 1.18–17.31; and OR 3.87, 95% CI 1.18–17.31, respectively.

**Conclusion:**

Upregulation of SLC7A3, SLC7A5, and SLC7A11 expression was associated with poor prognosis in PTC patients, and SLC7 gene expression levels are potentially useful prognostic biomarkers.

## Background

PTC has become the fifth most common cancer in women in the USA, and the incidence rate continues to increase worldwide [1]. PTC consists of four main histologic subtypes, including papillary thyroid carcinoma (PTC), follicular, anaplastic, and medullary thyroid cancer. PTC patients typically exhibit a favorable prognosis compared with follicular thyroid carcinoma patients [2]. However, PTC occasionally dedifferentiates and becomes aggressive and lethal. Therefore, the identification of key prognostic biomarkers and effective druggable targets is critical to the survival of PTC patients.

The solute carrier (SLC) 7 family genes comprise 14 genes. The gene family can be divided into two subgroups, the cationic amino acid transporters (SLC7A1-4) and glycoprotein-associated transporters (SLC7 family 5-14) [3]. Tumor cells are highly demanding for nutrients to support their fast growth rate. Therefore, amino acid transporters are essential for the maintenance of amino acid nutrition and survival of tumor cells [4]. To date, two amino acid transporters have been found to be over-expressed in cancers: SLC7A5 and SLC7A11. SLC7A5 is highly expressed in most cancers [5]. The hypoxia-inducible factor HIF2a upregulates SLC7A5 [6], which plays a critical role in cancer growth and progression. SLC7A5 promoter has canonical binding sites for the oncogene c-Myc, and overexpression of the oncogene causes upregulation of SLC7A5 [7]. Increased expression of SLC7A11 is observed in a variety of cancers where the transporter-assisted promotion of glutathione synthesis reduces oxidative damage and protects the cancer cells from apoptosis [8].

The associations among mortality, clinicopathologic characteristics, and SLC7 family genes remain unknown in PTC. To address these issues, the present study investigated the relationship between mortality and clinicopathologic characteristics, mortality and SLC7 family gene expression, clinicopathologic characteristics and SLC7 family gene expression by analyzing a large set of PTC patient data from The Cancer Genome Atlas (TCGA) database [9].

## Materials and methods

### Data acquisition

Data used in the study included normalized SLC7 family gene mRNA expression data and various clinical data from 355 PTC patients. Data were downloaded from the TCGA database [9] (http://firebrowse.org/?cohort=THCA&download_dialog=true). The clinicopathologic characteristics analyzed in the study included patients’ age, gender, thyroiditis, tumor size, extrathyroidal extension (ETE), American Joint Committee on Cancer (AJCC) stage, survival status, and follow-up data (months).

BRAFV600E mutational status is associated with recurrence and worse prognosis in PTC patients [10–12]. RAS mutations may confer a more aggressive phenotype in some PTC cases, increasing a patient’s risk for tumor recurrence, distant metastases, and death [13, 14]. Therefore, to analyze the associations between SLC7 family gene expression and BRAF and RAS mutation status, we downloaded somatic mutations of BRAF and RAS genes from the TCGA database. PTC patients were divided into BRAF- or RAS-mutant and wild-type groups based on their mutation status.

### Correlations between clinical characteristics, mortality, and expression of SLC7 family genes

In order to characterize the associations between clinical features and mortality, Student’s *t* test was applied to compare the age and tumor size between PTC patients who were deceased versus alive. Fisher’s exact test was used to analyze the associations between survival status and patients’ gender, thyroiditis, ETE, AJCC cancer stage, BRAF, and RAS mutation status. Linear regression models were used to evaluate the associations between clinical features and SLC7 family gene expression. For correlation coefficients, *t* values were extracted. Pearson’s correlation was conducted between different SLC7A mRNA expressions in PTC. All statistical analyses were conducted in R (version 3.2.0), and *P* < 0.05 was considered statistically significant.

### Survival analyses

To characterize the association of SLC7 family gene expression with patient survival, Patients were assigned to the “high-expression” group if they exhibited SLC7 family gene expression levels greater than the median values, whereas those patients exhibiting expression levels less than the median values were assigned to the “low-expression” group. Kaplan-Meier survival analysis was performed to plot survival curves, and the log-rank test was utilized to compare the difference in survival rates between the high- and low-expression groups using the R package of survival [15, 16]. Univariate and multivariate survival analyses were performed using logistic regression model. *P* < 0.05 was considered statistically significant.

## Results

### General characteristics of 355 PTC patients

The age of 355 PTC patients range from 15 to 88 years old, with the mean of 46.48. The average tumor size was 3 cm. Two hundred fifty-eight patients are female and 97 patients are male. One hundred sixteen and 118 PTC patients had thyroiditis and extrathyroidal extension respectively. One hundred ninety-five cancer samples had BRAF mutations, while 160 samples were wild-type. Twenty-four and 331 cancer samples were RAS-mutant or wild-type respectively. At the last day of follow-up, 14 PTC patients were deceased, and 341 were alive. The mean follow-up was 34.94 months (interquartile range, 14.31–43.87 months).

### The association between mortality and clinicopathologic characteristics in PTC

**Fig. 1:**
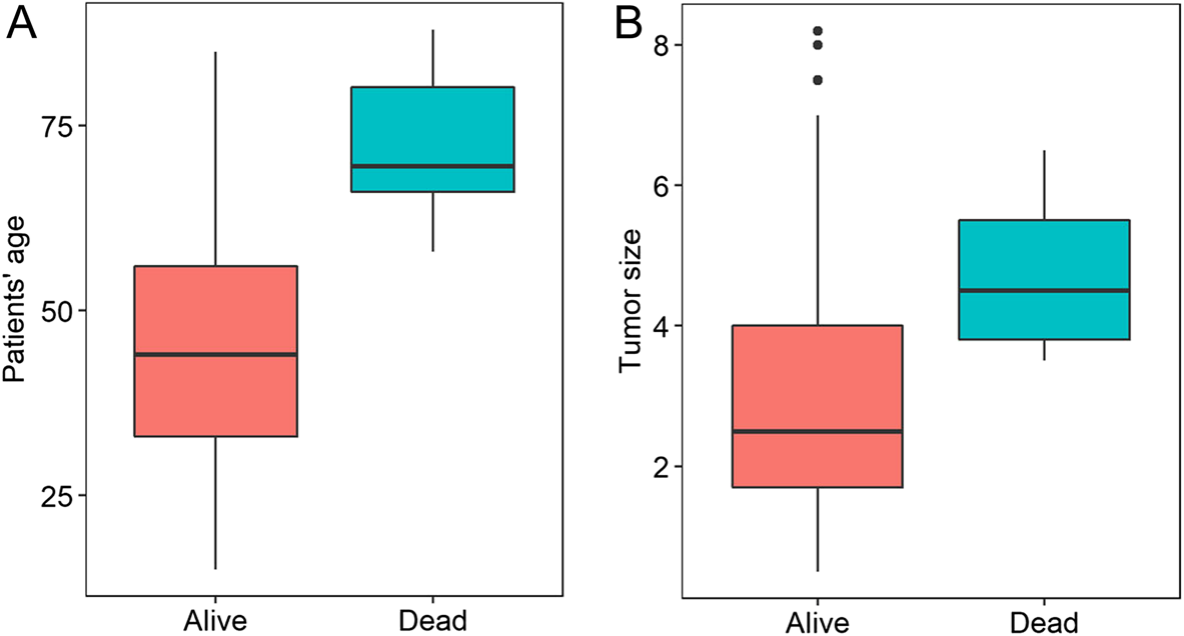
Comparison of patients’ age (**a**) and tumor size (**b**) between PTC patients who were deceased or alive

Among the clinicopathologic characteristics, patients with older age or larger tumor sizes exhibited significantly worse mortality than those with younger age or smaller tumor sizes, respectively (*P* < 0.0001 for all cases, Student’s *t* test, Fig. 1a, b). Moreover, mortality was significantly associated with increased cancer stage and absence of thyroiditis in PTC patients (*P* < 0.05 for all cases, Fisher’s exact test, Table 1). However, no significant association was observed between patient mortality and gender, ETE BRAF, or RAS mutation status (*P* > 0.05 for all cases, Fisher’s exact test, Table 1).

**Table 1:** Association between the clinicopathologic characteristics and overall survival status

Variables	Group	Alive	Dead	*P* value
Gender	Female	249	9	0.54
	Male	92	5	
Thyroiditis	Absent	225	14	0.006
	Present	116	0	
Extrathyroid extension	No	231	6	0.09
	Yes	110	8	
AJCC TNM stage	I/II	234	2	< 0.0001
	III/IV	107	12	
BRAF status	Wild-type	155	5	0.62
	Mutant	186	9	
RAS status	Wild-type	317	14	0.61
	Mutant	24	0	

### The association between clinicopathologic characteristics and SLC7 family gene expression in PTC

A linear regression model was applied to characterize the associations between clinicopathologic characteristics and mRNA expression values of SLC7 family genes. Tumor size was negatively correlated with SLC7A3 mRNA expression. Thyroiditis was significantly correlated with SLC7A2, SLC7A3, SLC7A7, and SLC7A9 (Table 2). ETE was significantly associated with SLC7A3, SLC7A8, and SLC7A9. AJCC TNM stage was associated with expression of SLC7A3, SLC7A8, and SLC7A11 (Table 2). RAS mutation status was associated with SLC7A3, SLC7A4, SLC7A6, and SLC7A8. BRAF mutation status was associated with SLC7A1, SLC7A2, SLC7A4, SLC7A6, SLC7A8, SLC7A9, and SLC7A14. Mortality was positively associated with greater mRNA expression of SLC7A3 (*t* = 2.39, *P* = 0.02) and SLC7A5 (*t* = 2.02, *P* = 0.04) expression (Tables 2 and 3). The results demonstrated that SLC7A3 and SLC7A5 expression was significantly correlated with patient mortality in PTC.

**Table 2:** Linear regression analysis between clinicopathologic characteristics and normalized SLC7 family gene expression in thyroid cancer patients

Gene	Age	Tumor size	Gender (male)	Absence of thyroiditis	Absence of ETE	AJCC stage (III/IV)	BRAF mutation	RAS mutation	Mortality
SLC7A1							++		
SLC7A2				−			+++		
SLC7A3				++	+	++		−	+
SLC7A4							+++	−−−	
SLC7A5									+
SLC7A6							−−−	+++	
SLC7A7				+++					
SLC7A8	−				−−	−−	+	−−	
SLC7A9				++	−		−−−		
SLC7A10									
SLC7A11						−			
SLC7A13									
SLC7A14							−		

*+*, positive correlation with *P* value < .05; *++*, positive correlation with *P* value < .01; *+++*, positive correlation with *P* value < .001

−, negative correlation with *P* value < .05; −, negative correlation with P value < .01; −−, negative correlation with *P* value < .001

**Table 3:** Linear regression analysis between clinicopathologic characteristics and mRNA expression count of SLC7A3, SLC7A5, and SLC7A11 genes

Clinical features	SLC7A3		SLC7A5		SLC7A11	
	*t* value	*P* value	*t* value	*P* value	*t* value	*P* value
Age	0.76	0.45	− 1.61	0.11	− 1.04	0.30
Tumor size	0.49	0.63	− 0.95	0.34	− 0.51	0.61
Gender (male)	3.18	0.00	0.93	0.35	− 1.34	0.18
Thyroiditis	−0.92	0.36	0.61	0.54	1.17	0.24
ETE	2.17	0.03	0.85	0.39	− 1.62	0.11
AJCC stage	2.69	0.01	− 1.01	0.31	− 2.06	0.04
BRAF mutation	− 1.49	0.14	0.42	0.67	− 0.92	0.36
RAS mutation	− 2.41	0.02	− 1.89	0.06	0.48	0.63
Mortality	2.39	0.02	2.02	0.04	1.65	0.10

### The correlations of expression of SLC7 family genes

**Fig. 2:**
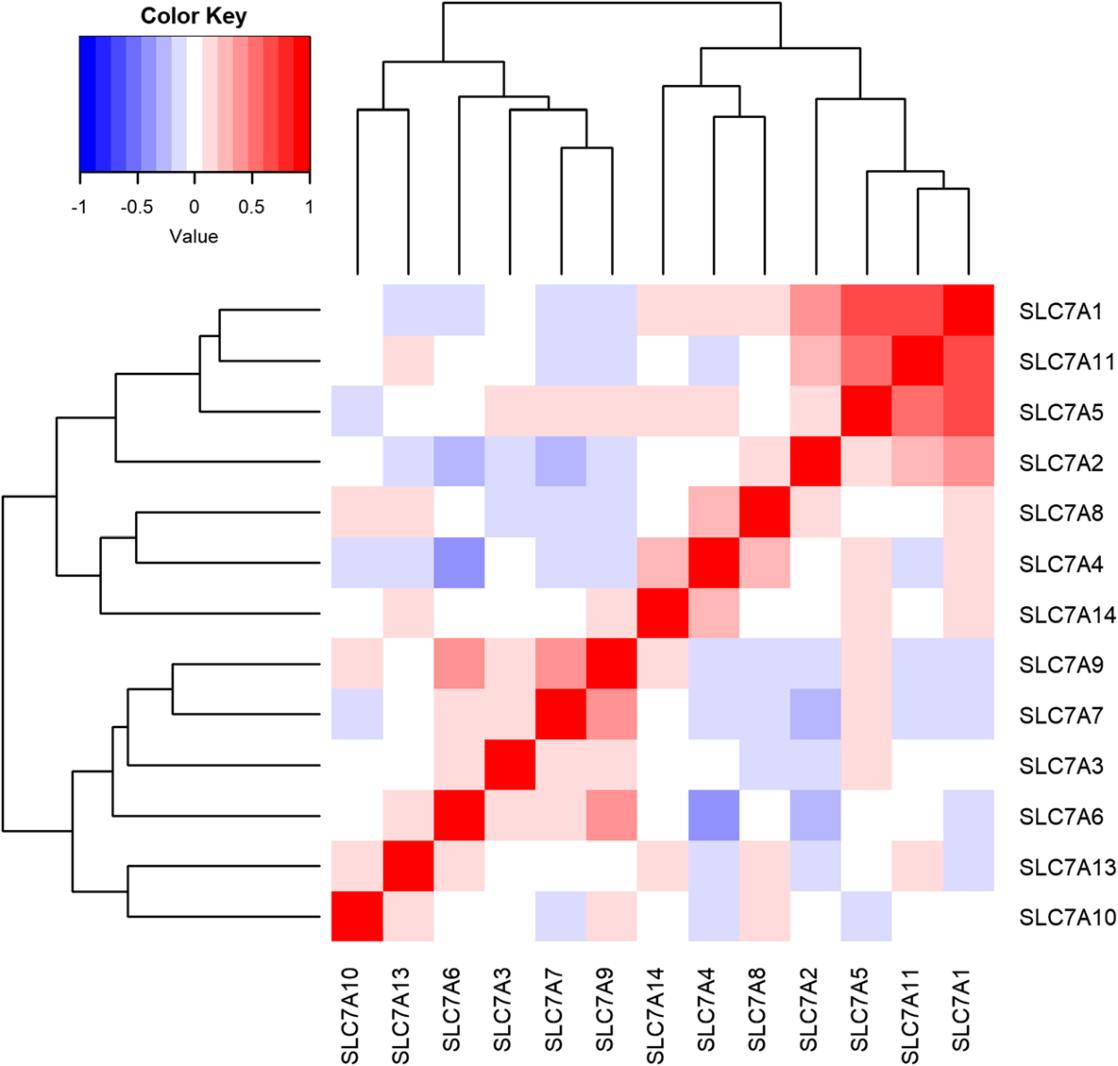
Correlations of expression of SLC7 family genes in 355 PTC patients

In order to characterize the correlations between different SLC7A mRNA expressions in PTC, the correlations of expression of SLC7 family genes were analyzed. The expression of SLC7 family genes was highly correlated, with SLC7A1 and SLC7A9 expression most frequently correlated with other SLC7 family members (*P* < 0.05 for all cases, Pearson’s correlation, Fig. 2). The expression of SLC7A3 was significantly correlated with the expression of SLC7A5, SLC7A7, SLC7A8, and SLC7A9. SLC7A5 expression showed significant correlation with SLC7A1, SLC7A2, SLC7A3, SLC7A4, SLC7A7, SLC7A11, and SLC7A14 genes expression. The expression of SLC7A11 was correlated with SLC7A1, SLC7A2, SLC7A5, SLC7A7, SLC7A9, and SLC7A13 with significant evidence (*P* < 0.05 for all cases, Pearson’s correlation, Fig. 2).

### Survival analyses between patient mortality and SLC7 family gene expression in PTC

**Fig. 3:**
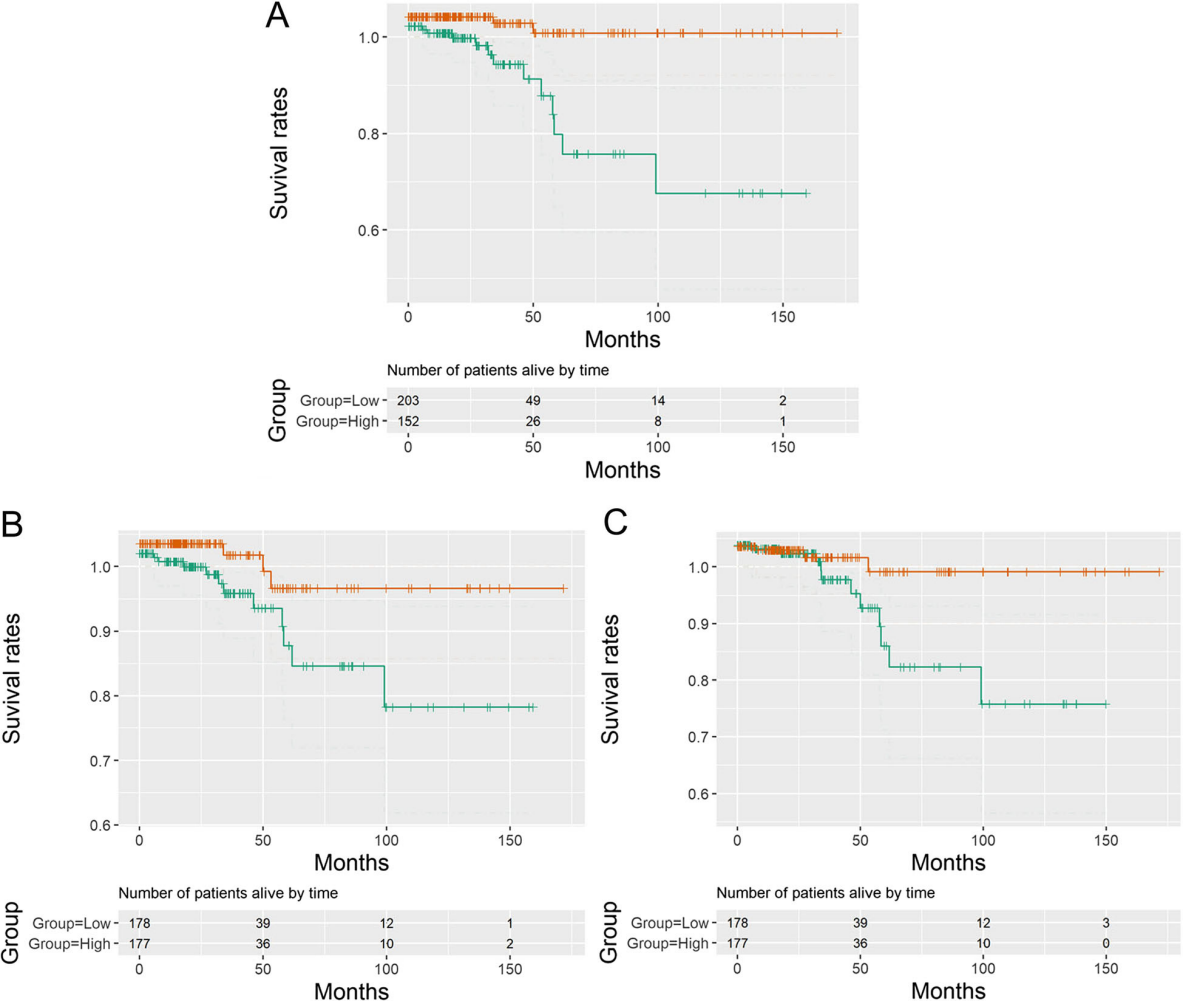
Kaplan-Meier survival analysis between patients’ clinical outcomes and SLC7A3 (**a**), SLC7A5 (**b**), and SLC7A11 (**c**) gene expression levels in 355 PTC patients

To evaluate the association of SLC7 family gene expression with patient survival, the 355 PTC patients were divided into low- and high-expression groups based on the median values. Kaplan-Meier survival analysis indicated that patients with high SLC7A3, SLC7A5, and SLC7A11 expression levels exhibited poorer survival than those with low SLC7A3, SLC7A5, and SLC7A11 expression levels (*P* < 0.05 for all cases, log rank test) (Fig. 3a–c). Univariate analysis using logistic regression model showed that SLC7A3, SLC7A5, and SLC7A11 were associated with increased mortality (*P* = 0.005, OR 8.61, 95% CI 2.3–55.91; *P* = 0.04, OR 3.87, 95% CI 1.18–17.31; and *P* = 0.04, OR 3.87, 95% CI 1.18–17.31, respectively.

To further validate the association of patients’ survival with SLC7A3, SLC7A5, and SLC7A11 expression levels, multivariate analyses were applied between patients’ survival and the mortality-associated features, including patients’ age, tumor size, cancer stage, thyroiditis, and SLC7A3, SLC7A5, and SLC7A11 expression levels. Multivariate survival analyses confirmed that SLC7A3, SLC7A5, and SLC7A11 were associated with increased mortality (*P* = 0.03, OR 12.13, 95% CI 1.65–256.97; *P* = 0.09, OR 4.56, 95% CI 0.88–34.73; and *P* = 0.03, OR 7.36, 95% CI 1.4–60.47, respectively).

## Discussion

In the present study, we have investigated the associations between mortality and clinicopathologic characteristics, mortality and SLC7 family gene expression, clinicopathologic characteristics, and SLC7 family gene expression in PTC. Many of SLC7 family members are significantly correlated to clinical traits of PTC patients. In particular, we found three genes, SLC7A3, SLC7A5, and SLC7A11, that showed significant associations with mortality, which might have clinical values. The gene SLC7A3 is a sodium-independent cationic amino acid transporter, which is glycosylated and localized to the plasma membrane. SLC7A3 is selective for cationic l-amino acids transportation [17]. So far, there is no report regarding the involvement of SLC7A3 in cancers, for the first time, our study found that SLC7A3 expression was associated with ETE, higher cancer stage, BRAF, RAS mutation, and mortality in PTC. Though the molecular mechanism on how SLC7A3 expression impacts mortality is unknown, SLC7A3 expression was independently associated with the RAS mutation, which is a well-known negative prognostic factor in PTC. This might in part explain the reasons; however, it still needs further studies.

Among the 14 SLC7 family genes, SLC7A5 has been most widely investigated in various cancers. SLC7A5 functions as an l-type amino-acid transporter that transports large neutral amino acids [18] and is over-expressed in many cancer types, such as prostatic, esophageal, gastric, and pancreatic carcinomas [19–22]. SLC7A5 is involved in the growth and mortality of a variety of tumor cells [18–23]. SLC7A5 expression is indicative of a poor prognosis in pancreas cancer [22, 24], melanoma [25], bile duct adenocarcinomas [26], and clear cell renal cell carcinoma [27]. Consistent with previously published results, our study demonstrated that high SLC7A5 expression was related to patient mortality in PTC.

SLC7A11 is a component of the cysteine/glutamate transporter, which plays a key role in glutathione synthesis. It has been evidenced that SLC7A11 has oncogenic functions in glioma. Overexpression of SLC7A11 resulted in decreased endogenous ROS levels as well as decreased migration and invasion in glioblastoma [28]. SLC7A11 expression is associated with accelerated growth and tumor-associated seizures [29] and predicts poor survival in patients with malignant glioma [29, 30]. In addition, over-expression of SLC7A11 increased resistance to oxidative stress and decreased chemosensitivity to temozolomide in the context of glioma [31]. For the first time, our study identified that SLC7A11 expression was associated with a poor prognosis in PTC, expanding the functions of SLC7A11 in tumorigeneses.

SLC7 family gene expression analysis might have clinical value in the near future. Cytologic or surgical specimens of PTC patients exhibiting high SLC7A3, SLC7A5, and SLC7A11 expression are expected to be associated with a poor prognosis. Therefore, more aggressive treatment or frequent follow-up may be recommended for these patients. Additionally, SLC7A3, SLC7A5, and SLC7A11 may represent novel and potential druggable targets in PTC. Drugs inhibiting SLC7A11 gene could have great therapeutic potential, as demonstrated by in vitro and vivo studies in breast cancer [32] and resistant head and neck cancer cells [33] using the SLC7A11 inhibitors.

Though this study provides the evidence that upregulation of SLC7A3, SLC7A5, and SLC7A11 genes is an indicator of poor overall survival in PTC patients, the study has certain limitation. The overexpression of SLC7A5 and SLC7A11 may be partly attributed to macrophages [34, 35], and tumor-associated macrophages are activated and present in thyroid cancer microenvironment [36]. Thus, not the mRNA expression of SLC7A5 and SLC7A11 but the presence of TAM in PTC tissue is the prognostic marker, this possibility needs further studies.

## Conclusion

In conclusion, upregulation of SLC7A3, SLC7A5, and SLC7A11 genes is associated with poor overall survival in PTC patients. SLC7 family gene expression represents a potentially prognostic biomarker to predict survival in PTC.

## Abbreviations

AJCC: American Joint Committee on Cancer
CI: Confidence interval
ETE: Extrathyroidal extension
OR: Odd ratio
PTC: Papillary thyroid carcinoma
TCGA: The Cancer Genome Atlas
